# Automated ERCC1 immunochemistry on hybrid cytology/tissue microarray of malignant effusions: evaluation of antibodies 8F1 and D-10

**DOI:** 10.1186/2043-9113-1-25

**Published:** 2011-09-30

**Authors:** Alex Soltermann, Sandra Kilgus-Hawelski, Silvia Behnke, Martina Storz, Holger Moch, Beata Bode

**Affiliations:** 1 Institute of Surgical Pathology, University Hospital Zurich, Schmelzbergstrasse 12, CH-8091 Zurich, Switzerland

## Abstract

**Background:**

The excision repair cross-complementation group 1 (ERCC1) protein is the key enzyme of the nucleotide excision repair (NER) pathway. Loss of protein expression on immunohistochemistry is predictive for platinum-based chemotherapy response. Frequently, the diagnosis of malignancy is made on cytologic effusion samples. Therefore, we evaluated the staining quality of monoclonal anti-ERCC1 antibodies 8F1 and D-10 on microarrays of malignant pleural and peritoneal effusions by automated immunochemistry.

**Methods:**

Cores from effusion cell blocks of 117 patients with > 40 malignant cell clusters per whole section (pleural n = 75, peritoneal n = 42) were assembled together with 30 histologic control cores from large tissue blocks (lung, breast and ovarian carcinoma, each n = 10) on hybrid cytology-tissue microarrays (C/TMA). Four immunochemistry protocols (Mab 8F1 and D-10, CC1-mono Ventana and H2-60 Bond automat) were performed. Immunoreactivity was semi-quantitatively scored for intensity and intensity multiplied by percentage staining (H-score).

**Results:**

Tumors were classified into female genital tract carcinoma (n = 39), lung adenocarcinoma (n = 23), mesothelioma (n = 15), unknown primary (n = 14), breast carcinoma (n = 10), gastro-intestinal carcinoma (n = 12) and other (n = 4). On both platforms, reproducible nuclear ERCC1 immunoreactivity was achieved with both antibodies, although D-10 was slightly weaker and presented more background staining as well as more variation in the low expression range. No significant differences were found between cytologic and histologic cores. Using the 8F1 CC1-mono protocol, lung and breast carcinomas had lower ERCC1 expression in comparison to the other entities (p-value < 0.05).

**Conclusions:**

Cytology microarrays (CMA) are suitable for investigation of clinical biomarkers and can be combined with conventional TMA's. Dichotomization of ERCC1 immunoreactivity scores is most suitable for patient stratification since definition of negativity is antibody-dependent.

## Background

Platinum-containing drugs like cisplatin are widely used in chemotherapy (CT) regimens of advanced cancers such as ovarian or lung carcinoma due to their robust effectiveness. Cisplatin forms DNA adducts, thereby causing inter- and intra-strand cross links, comparable to alkylating agents. If not repaired, this DNA damage will lead to apoptotic cell death or mutation. The cross links are removed by trans-lesion synthesis via nucleotide excision repair (NER), which is the primary repair system for bulky DNA lesions caused by such drugs [1]. In the NER system, the heterodimer ERCC1-XPF functions as a structure-specific endonuclease to make the 5'-incision on the damaged strand. This step is claimed to be the key factor [1-3]. Subsequently, a short oligonucleotide fragment containing the offending lesion is replaced. It was deduced that tumors with low nuclear ERCC1 expression better respond to platinum-containing CT because of reduced repair capability for DNA adducts [4,5]. Conversely, patients having tumors with high ERCC1 expression and thus functional NER and also HRR (homologous recombination repair) systems were found to have a better overall survival, since such tumors are assumed to be less unstable and dedifferentiated (so-called ERCC1 paradoxon). Thus, ERCC1 is considered an important predictive biomarker for response to platinum-containing CT. A valid predictor of this widely used regimen is of high clinical importance, because response rates in e.g. unselected non-small cell lung cancer (NSCLC) patients range from only 16 to 30% [6,7].

Assessment of tumoral ERCC1 expression has been performed in different settings, including preclinical, adjuvant and palliative studies [8,9]. The results of these studies were controversial. First, differences between mRNA and protein-based studies as well as between formalin-fixed, paraffin-embedded (FFPE) and frozen tissue were observed [10]. Second, protein expression was mostly assessed by immunohistochemistry (IHC) on FFPE tissue, using the mouse monoclonal anti-ERCC1 antibody clone 8F1 [11-14]. However, specificity and intranuclear compartmentalization of this clone was recently challenged [15,16]. The ERCC1 predictor concept is now at the point where profound and controlled validation in multi-centre ring-tests be envisaged since this biomarker is used as stratification parameter in oncologic trials. Thus tissue types, tissue processing and protocols of automated immunochemistry platforms need to be standardized.

Importantly, patients having advanced cancers, e.g. originating from ovary, lung or pleura, may primarily present with malignant peritoneal or pleural effusion. Often, the effusion is sent for cytologic diagnosis. Cytologic smears and cell blocks are prepared. No further tissue biopsy may be performed if patients are palliative. Thus, predictors such as EGFR (epidermal growth factor receptor) or ERCC1 are increasingly demanded by clinicians on cytologic material. There are although relevant technical differences between histology and cytology: Histologic sections are 2 to 4 μm thick, therefore only a part of the tumor cell nucleus is represented since e.g. NSCLC nuclei have by definition a diameter > 30 μm (> 3 × resting lymphocyte diameter) [17]. In contrast, on cytologic smears, entire tumor cells are adherent to the glass slide, thus nuclei are conserved in all 3 dimensions, including z-axis. This fact may lead to major differences when counting nuclear EGFR signals by fluorescence in-situ hybridization (FISH) or semi-quantitatively scoring protein expression intensities by immunohistochemistry. Manufacture of cytologic cell blocks out of the sediment is a means to circumvent cyto-histologic discrepancies since cut thickness is equal.

We have previously investigated the 3 anti-ERCC1 antibodies Mab 8F1, Mab D-10 and Rab FL-297 on a retrospective NSCLC patient cohort assembled on a tissue microarray (TMA) [18]. Only 8F1 and D-10 could be confidently scored. The rabbit polyclonal ab FL-297 presented high cytosolic background and rare weak nuclear staining, thus was omitted. In this study, we aimed for evaluating the staining quality of the 8F1 and D-10 antibodies on cytologic effusion cell blocks from most common cancers associated with malignant effusions. Cores from cell blocks and histologic controls were assembled on two hybrid cytology/tissue microarrays (C/TMA) and immunochemistry performed on 2 different automated IHC platforms. We tested the null hypothesis that both antibodies yield similar staining performance due to consistent cut thickness of 4 μm across the whole C/TMA surface.

## Methods

### Patient cohort

Cytologic cell blocks of malignant pleural or peritoneal effusions of 125 patients in the time frame 2005-2010, presenting high amounts of malignant cells (> 40 clusters per whole section surface) were enrolled in the study. Following diagnostic categories were set up based on morphology, clinical data and immunochemistry with respective markers (using e.g. TTF-1 or Ber-EP4 in case of differential diagnosis between lung adenocarcinoma and mesothelioma): Female genital tract carcinoma (including ovarian, primary peritoneal and uterine carcinoma), lung adenocarcinoma, mesothelioma, breast carcinoma, gastro-intestinal carcinoma (including pancreas, colon and oesophagus carcinoma), unknown primary tumor and other (including squamous cell and large cell carcinoma of the lung, transitional carcinoma of the bladder and rhabdomyosarcoma). On a first C/TMA, 56 tumoral cell block cores were assembled, together with controls (n = 16) including benign inflammatory-reactive pleural effusions and histologic tissues from mesothelioma, adenocarcinomas of different organs, transitional cell carcinoma of the bladder and a thoracic lymph node. These controls were not computed. On a second C/TMA, 69 tumoral cell block cores were assembled together with non-matched control histologic cores from lung, breast and ovarian carcinomas (each n = 10, total n = 30). During processing, malignant cells were lost or immunochemistry was incomplete, respectively, in 8 of 125 cases, thus 117 tumoral cytologic cell block cores from both C/TMAs and all 30 histologic controls from the second could be entirely scored. The study was approved by the institutional review board of the University Hospital Zurich (reference number StV 29-2009).

### Cell block

The effusion liquid was centrifuged at 2000 × g for 10 min at room temperature and the cell-free supernatant discarded, leaving a small amount of 100 μl liquid above the sediment. The sediment consisted of an upper white phase, containing the tumor cells as well as lymphocytes and mesothelial cells. The lower red phase represented erythrocytes. The upper white phase was aspirated with a Pasteur pipette and few droplets used for manufacture of 3 Papanicolaou stained smears. The rest of the white phase was then transferred into a microtube. A clot was quickly formed by addition of 4 droplets plasma (from the hospital's blood donation service) and 1 droplet thrombin (60 NIH-U/ml, Diagnotec AG, Liestal, Switzerland). The clot was transferred into a small inlay cassette with a wooden stick and this cassette was put into a larger histology cassette. After formalin fixation, clots were processed by paraffin embedding and haematoxylin-eosin (H&E) staining of whole sections.

### Hybrid cytology/tissue microarray

From a representative region of the donor block, a paraffin core of 0.6 mm diameter and 3-4 mm height was taken and precisely arrayed into a new recipient paraffin block using a custom-made, semiautomatic tissue arrayer (Beecher Instruments, Sun Prairie, WI, USA). Four μm sections were cut for immunochemistry.

### Immunochemistry

The two mouse monoclonal anti-ERCC1 antibodies 8F1 (Novus Biologicals, Littleton, CO, USA, dilution 1:30) and D-10 (Santa Cruz Biotechnology, Santa Cruz, CA, USA, dilution 1:100), directed against full length protein, were tested on 3 multi-tissue microarrays to select the appropriate dilution as described [18] and further evaluated on whole sections of NSCLC for surface homogeneity. Two automated immunochemistry platforms were used: First, on a Ventana Benchmark^® ^platform (Ventana Medical Systems, Tucson, AZ, USA), the cell conditioner 1 (CC1) standard mono protocol (CC1-mono) was performed: pre-treatment with boiling for 60 min in pH 8 Tris buffer, incubation with primary ab for 60 min at room temperature (RT) and development with the Ultraview-HRP mono kit, including incubation with respective secondary ab for 30 min at RT and additional amplification with respective third and fourth ab. Second, on a Leica Bond^® ^platform (Vision Biosystems, Melbourne, Australia), the H2 standard (H2-60) protocol was performed: pre-treatment with boiling for 60 min in pH 8 Tris buffer, incubation with primary ab for 30 min at RT and subsequent development with the Refine-DAB Bond kit, including incubation with respective secondary ab for 30 min at RT and additional polymer amplification. For TTF-1, the monoclonal antibody SPT24 (Novocastra Laboratories Ltd, Newcastle upon Tyne, UK, dilution 1:100) was used with protocol Ventana CC1-mono. For Ber-EP4 we used Mab M0804 (DakoCytomation, Baar, Switzerland, dilution 1:40) with protocol prediluted protease 1 Ventana and 4 min digestion.

### Scoring system

Nuclear immunoreactivity of both the 8F1 and the D-10 ab was scored by A.S. in a blinded manner. The staining intensity was semi-quantitatively scored 0 (negative), 1 (weak), 2 (moderate) or 3 (strong). Further, the percentage of cells having any positivity was proportionally scored 0 (0%), 0.1 (1-9%), 0.5 (10-49%) or 1.0 (50% and more) as described [4]. The H-score was obtained by multiplication of intensity with percentage staining (final range 0 to 3, per core). Endothelial cells in lymphatic control tissue were assigned an intensity of 2 by default.

### Image capture and statistical analysis

Images were captured on a Zeiss Axioskop connected to a CCD camera, using the image analysis software analySIS FIVE (Olympus BioSystem, Volketswil, Switzerland). White balance was adjusted on analySIS FIVE. No further image processing on Adobe Photoshop such as application of gradation curves for enhancement of contrast or brightness was performed. Correlations of ERCC1 immunoreactivity scores with tumor entities were computed using non-dichotomized data and Kendall's tau-b tests, comparison of score means by the Mann-Whitney U test. P-values < 0.05 were considered significant. Analyses were carried out on PASW 18.0.0 software package (SPSS Inc., Chicago, IL, USA).

## Results

### Cohort description

Of the 117 patients (pleural effusion n = 75, peritoneal n = 42) 77 were female and 40 male. The mean age was 66 years (range 29 to 91 years). Table 1 indicates the frequencies of each diagnostic category in both C/TMAs. We concluded that this distribution well represents most common cancers giving rise to malignant effusions and thus is adequate for further investigations.

**Table 1:** Overview of tissue cores assembled in the 2 C/TMAs, including controls from reactive effusions and histologic solid tumors.

	C/TMA 1	C/TMA 2	Total	
	n	n	n	%
**Tumoral cell blocks**				
Female Genital Tract Ca	13	26	39	33.3
Lung Adeno Ca	12	11	23	19.7
Mesothelioma	6	9	15	12.8
Unknown Primary	12	2	14	11.9
Gastro-Intestinal Ca	3	9	12	10.3
Breast Ca	3	7	10	8.6
Other	4		4	3.4
Total	53	64	117	100

**Control cell blocks**				
Reactive pleural effusion	5			
Total	5			

**Control histology**				
Female Genital Tract Ca	2	10		
Lung Adeno Ca	1	10		
Breast Ca	1	10		
Mesothelioma	1			
Oesophagus Ca	1			
Colon Ca	1			
Lung Squamous Cell Ca	1			
Prostata Ca	1			
Bladder Ca	1			
Thoracic Lymph Node	1			
Total	11	30		

### ERCC1 protein expression on whole sections

**Figure 1:**
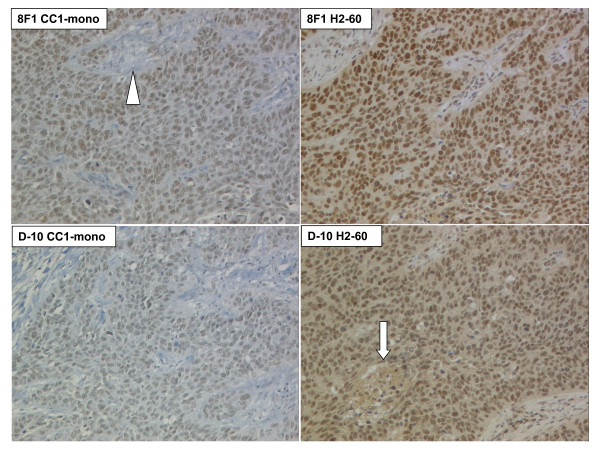
**Anti-ERCC1 immunohistochemistry on whole sections of a lung squamous cell carcinoma, using Mab 8F1 and D-10 with CC1-mono and H2-60 protocols**. Note increased cytosolic background with H2-60. Arrow: Necrotic centre. Arrowhead: Stromal axis. 100 × original magnification.

In order to check for surface homogeneity of immunoreactivity, we first stained 4 μm thick whole sections of squamous cell lung carcinoma (Figure 1). No image processing such as enhancement of contrast or brightness was performed, except adjustment of white balance. Distinct nuclear staining was achieved with all 4 protocols; however intensity and background varied significantly. Intensity was higher for both antibodies with the H2-60 protocol, although on the cost of increased cytosolic background. The CC1-mono protocol yielded weaker staining, particularly for D-10, but no background. Homogeneous staining was observed over the entire tissue surface. Nuclei were equally stained and no intranuclear compartmentalization was visible apart from omission of nucleoli or nuclear invaginations. Few stroma and necrosis (< 25% of total surface) was present on the respective whole section, but contributions of immunoreactivity from these compartments were negligible. We concluded that such a surface would be amenable to automated quantitative intensity measurements including creation of a continuous variable.

### ERCC1 protein expression on hybrid C/TMA sections

**Figure 2:**
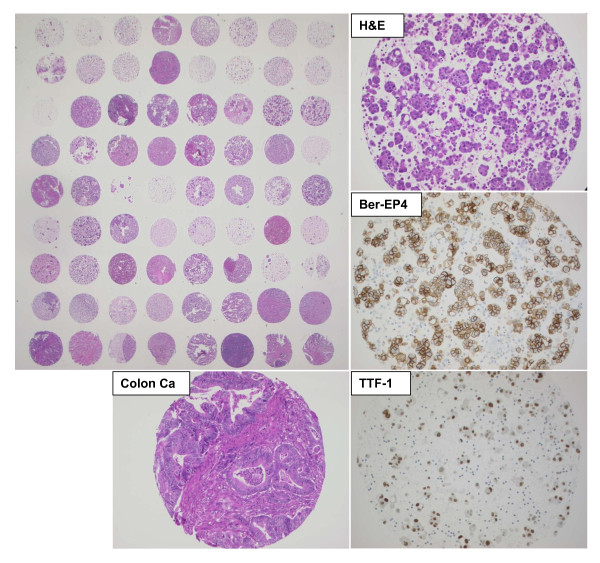
**Whole section view of first hybrid C/TMA and representative core from cytologic cell block of a lung adenocarcinoma, stained with H&E, Ber-EP4 and TTF-1**. Lower left: control histologic core of a colon adenocarcinoma. Compare cellular density and thickness of tissue between cytologic and histologic core.

**Figure 3:**
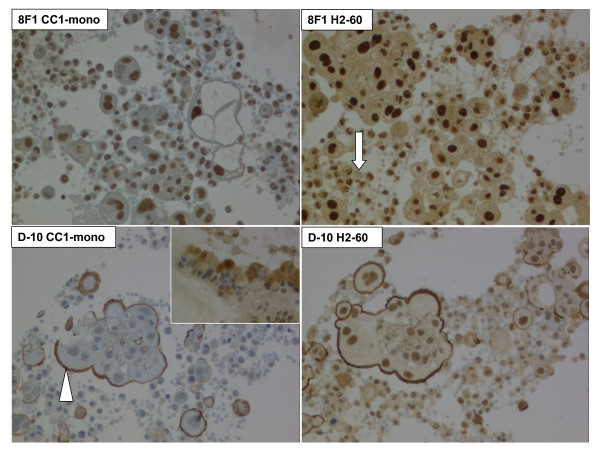
**Anti-ERCC1 immunocytochemistry on cell block core of malignant pleural mesothelioma, using Mab 8F1 and D-10 with CC1-mono and H2-60 protocols**. Arrow: Surrounding non-tumoral cells, including lymphocytes, macrophages and neutrophil granulocytes. Arrowhead: Unspecific plasma membrane staining with D-10. 200 × original magnification. Inset lower left: Staining of intracellular mucin vacuoles of a mucinous adenocarcinoma of unknown origin. 400 × original magnification.

**Figure 4:**
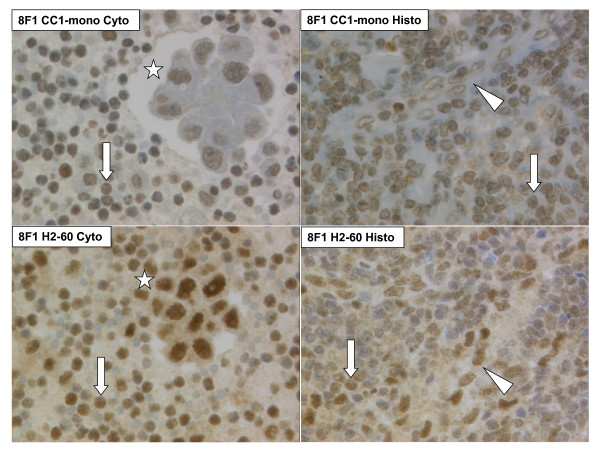
**Cyto-histologic comparison of anti-ERCC1 immunoreactivity using the protocol 8F1 CC1-mono (top) or 8F1 H2-60 (bottom)**. Left: Pleural effusion sediment of lung adenocarcinoma. Right: thoracic lymph node. Arrow: Lymphocyte. Arrowhead: Streak of endothelial cells. Asterisk: Tumor cell cluster. 400 × original magnification.

Consecutive C/TMA sections were first stained for H&E and respective diagnostic markers. Representative images are presented on Figure 2. Second, the four anti-ERCC1 protocols were performed. Both antibodies yielded distinct nuclear signals, although D-10 presented additional focal, but strong background staining of the plasma membrane and intracellular mucin vacuoles (Figure 3). Importantly, many tumor cell clusters were heavily admixed with inflammatory cells also expressing ERCC1 with intensity score 2-3. Inflammatory cells of both malignant and benign effusion sediments stained equally intense compared to lymphatic parenchyma on histologic cores (Figure 4). The same was observed for intratumoral inflammatory infiltrates of histologic solid tumors (data not shown). Further, the cores from the patients with non-malignant control effusions all had reactive mesothelial cells again expressing ERCC1 score 2-3. We concluded that ERCC1 staining intensity of such surfaces is difficult to be quantitatively measured since up to 50% of immunoreactivity is due to surrounding reactive and inflammatory cells.

### Distribution of intensity and H-scores

For practical reasons, ERCC1 protein expression levels have been dichotomized in most publications closest to the median into low/high, although alternative cut-off's were tested [4,19], since the definition of "ERCC1 negative" is pending. To address this issue, the statistical distributions of the ERCC1 scores were analysed as following: ERCC1 means were consistently found to be in the 1.3 to 2.3 range for all protocols; D-10 antibody incubated with the CC1-mono protocol defining the lower end (Table 2, part A). No significant differences regarding means were found between intensity only and intensity multiplied by percentage of positive cells (H-score). Comparing cytologic versus histologic cores, the average of all 8 means was slightly lower in controls, but this was not significant (p-value 1.000, Mann-Whitney U test).

**Table 2:** Summary of statistical data.

		8F1				D-10				
		CC1-mono		H2-60		CC1-mono		H2-60		
		Int	H	Int	H	Int	H	Int	H	Average
**A. Score means**										
**Cell blocks**		2.10	2.06	2.25	2.18	1.51	1.28	2.20	2.19	1.97
**Histo controls**		1.63	1.57	1.97	1.92	1.63	1.46	2.23	2.08	1.81

**B. Distribution %**										
**Cell blocks**										
0		1.7	1.7	3.4	3.4	12.8	12.8	0.0	0.9	4.6
0.1		0.0	0.9	0.0	4.3	0.0	19.7	0.0	0.9	3.2
0.5		0.0	1.7	0.0	2.6	0.0	10.2	0.0	5.0	2.4
1		21.4	21.4	13.7	7.6	42.7	13.7	17.9	12.8	18.9
1.5		0.0	0.0	0.0	0.0	0.0	0.0	0.0	0.9	0.1
2		41.9	39.3	37.6	36.8	24.8	23.9	44.5	42.7	36.3
3		35.0	35.0	45.3	45.3	19.7	19.7	37.6	36.8	34.3
Total		100.0	100.0	100.0	100.0	100.0	100.0	100.0	100.0	100.0

**C. Score means**										
**Cell blocks**										
Lung Adeno Ca		1.96	1.94	2.22	2.26	1.43	1.35	2.13	2.03	1.92
Breast Ca		1.90	1.61	1.90	1.81	1.60	1.37	1.90	1.65	1.72
Female Genital Tract Ca		2.13	2.13	2.28	2.20	1.72	1.48	2.33	2.29	2.07
Mesothelioma		2.47	2.47	2.40	2.34	1.80	1.53	2.40	2.40	2.23
Gastro-Intestinal Ca		2.33	2.33	1.92	1.83	1.25	1.10	2.00	2.00	1.85
Unknown Primary		1.93	1.86	2.57	2.57	1.21	0.69	2.21	2.11	1.89
Other		1.75	1.63	2.00	2.00	0.50	0.25	1.75	1.63	1.44
**Histologic controls**										
Lung Adeno Ca		1.10	0.90	1.50	1.40	1.20	0.78	1.70	1.40	1.25
Breast Ca		1.90	1.90	2.00	1.95	1.60	1.50	2.50	2.35	1.96
Female Genital Tract Ca		1.90	1.90	2.40	2.40	2.10	2.10	2.50	2.50	2.23

**D. Correlat.ion entities**										
Lung AdenoCa	p	*0.019*	*0.009*	ns	ns	ns	ns	ns	ns	ERCC1 lower
Breast Ca	tau	0.186	0.201							Lung Adeno
Female Genital Tract Ca										and Breast Ca
Mesothelioma										
Gastro-Intestinal Ca										

A. Means of intensity and H-scores across cytologic (n = 117) and histologic (n = 30) cores. B. Distributions (%) of score values. C. Means of scores among tumor entities on both cytologic and histologic cores. D. Correlation of ERCC1 scores with tumor entities (Kendall's tau-b test used).

Regarding distributions of possible score values in %, a potential advantage of the H-score was again scarcely visible (Table 2, part B). Consider e.g. the D-10 ab with protocol CC1-mono: Multiplication with percentage of positive cells results in more degrees of freedom, but with regard to dichotomization closest to the median, both intensity and H scores need to be equally dichotomized 0 to 1 (55.5 and 56.4% low, respectively) against 2 to 3 (44.5 and 43.6% high, respectively). Importantly, truly accepting only score 0 and 0.1 as "ERCC1 negative" would mean that for the D-10 antibody from zero (H2-60 protocol, intensity score) to 32.5% (CC1-mono protocol, H-score) of tumors are negative and thus primarily suited for cisplatin-containing chemotherapy. However, for the 8F1 antibody much less variation was found in the low expression range. Further, using a dichotomization of 0 to 1 versus 2 to 3, from 43.6% (D-10 ab, CC1-mono, H-score) to 82.9% (8F1 ab, H2-60, intensity score) of tumors would be classified as ERCC1 high. We concluded that statistical distributions of ERCC1 protein expression levels are dependent on technical aspects, in particular selection of antibody and incubation protocol.

### Correlation with tumor entities and diagnostic markers

All ERCC1 scores were next computed against the tumor categories (Table 2, part C and D). Female genital tract carcinoma and mesothelioma had average score means above 2, whereas the categories breast carcinoma and other marked the lower end. Comparison of cytologic with histologic scores showed similar mean values for breast and female genital tract carcinoma, whereas the lung adenocarcinoma controls had lower expression of ERCC1 compared with cytologic cores. In more detail, we computed scores among lung adeno, breast, female genital tract and gastro-intestinal carcinoma as well as mesothelioma by Kendall's tau-b tests. The intensity and H-score of the 8F1 antibody with protocol CC1-mono were significantly lower in lung adenocarcinoma and breast carcinoma. However, this relation was not found with any of the other scores. We concluded that cells from most common malignancies giving rise to pleural or peritoneal effusion display a robust ERCC1 protein expression and that no particular entity has completely lost expression of this enzyme.

## Discussion

In this study, we have investigated the immunochemical performance of the 2 mouse monoclonal anti-ERCC1 antibodies 8F1 and D-10 on cell blocks of malignant pleural and peritoneal effusions assembled together with histologic control cores to hybrid C/TMAs.

Oncologic trials have started using the ERCC1 expression level as stratification parameter for inclusion into a respective study arm; therefore measurements must be reproducible. Several studies in the preclinical, adjuvant and palliative setting have been performed, using 2 main laboratory approaches: First, patient tumor tissue was examined for ERCC1 expression by either RT-PCR (mRNA) or IHC (protein). Second, tumor tissue or peripheral blood components were genotyped by PCR to examine for SNPs (single nucleotide polymorphism). However, resulting data (comprehensively reviewed in [8,9]) is conflicting and entirely opposite correlations were observed. Main reasons for these discrepancies may be differences between fresh frozen and FFPE tissue [10], the small size of bronchial biopsies comprising only few tumor cells and cohort bias due to histotype composition. ERCC1 expression is e.g. higher in squamous cell carcinoma compared to adenocarcinoma. Assessment of SNPs is a new method, mainly investigated in patients with advanced colorectal carcinoma treated with oxaliplatin. Again, e.g. the allelic combination T/T was associated on the one hand with a better RR (response rate), on the other hand with increased risk of progression [8].

For many patients with advanced cancer, only cytologic smears and corresponding sediments may be available. These sediments can be processed into paraffin cell blocks. The cell block technology has attracted much interest since serial sections, potentially > 100, can be manufactured and used for assessment of clinically relevant biomarkers, such as EGFR and EML4-ALK (echinoderm microtubule-associated protein-like 4; anaplastic lymphoma kinase) FISH (fluorescent in-situ hybridization) or DNA extraction for PCR of EGFR exons 18-21. Data from these cell block sections is highly comparable to corresponding sections of histologic tissue biopsies or surgical specimens due to the same cut thickness, in most laboratories 2 to 4 μm. Furthermore, paraffin cores of 0.6 mm diameter from sediment blocks or also cell line pellets can be assembled into a cytology microarray the same way than cores from histologic blocks into a tissue microarray [20-22]. A cell block may also be an effective quality assurance tool for cancer registries and national mortality statistics [23], since no further diagnostic procedures maybe performed if e.g. a positive pleural effusion defines the pM1a advanced stage of lung adenocarcinoma.

However, formalin fixation time between cytologic and histologic cores can be significantly different. Clots of tumor cells are quickly formed with plasma/thrombin and often fixed only during the day for some hours. The inlay cassette is then processed the same night on the fixation/staining automat. Conversely, surgical specimens are frequently fixed for up to 48 h before tissue cuts are loaded on the over-night automat. Thus the major question arises if cytologic cell blocks are usable the same way for biomarker assessment or if additional tissue biopsies need to be taken. Notably, such biopsies are taken only for biomarker investigation and are increasingly considered as integral part of translational research protocols. Ethical concerns have been raised for this strategy and some organs such as lung have not negligible intervention risk. On our hybrid C/TMAs we noticed that inflammatory cells in the effusion sediments had equal staining intensity compared to lymphatic parenchyma or intratumoral inflammatory infiltrate of solid tumors on histologic cores. Further, no significant differences in ERCC1 immunoreactivity were found between tumor cells in effusion liquid and solid sheets on histologic controls. Thus, potential influence of fixation time and depth of fixative penetration seems to be of minor importance.

Currently, immunocytochemistry can be performed on several types of effusion preparations: Ethanol-fixed smears, air-dried and post-fixed cytospins, liquid-based thin layers (ThinPrep), ethanol-fixed cell blocks and formalin-fixed cell blocks. Data on technique superiority is conflicting. Some authors observed best immunoreactivity with ethanol-fixed smears [24]; others experienced equal staining for non-nuclear but superior staining for nuclear markers for formalin-fixed cell blocks in comparison to ThinPrep slides [25]. In general, cell blocks seem to give better morphology and less background staining than cytospins or ThinPrep [26,27] and the use of a combined ethanol-formalin fixative has been reported to best preserve the cyto-morphologic features [28]. We thus believe that a formalin-fixation protocol is adequate for a nuclear epitope. Concerning embedding medium, agarose may be used as intermediate [29]. In our protocol a clot is formed by addition of plasma and thrombin to the cells. The question of optimal core diameter and minimal cellularity has been addressed [29,30]. The diameter of 0.6, 1, 2 or 3 mm defines the density on the glass slide, but core loss seems to be a minor problem with any diameter. In contrast, cellularity is of major importance when evaluating a larger antibody panel. The distinction into low (1 to 20 cell clusters), moderate (20 to 40 cell clusters) and high cellularity (> 40 cell clusters), one cell cluster being an aggregate ≥ 5 cells, seems reasonable and we have implemented the same concept, selecting only blocks with high cellularity. Concerning automated IHC/ICC platforms, the Bond protocol may yield a higher staining intensity due to an in-built polymer amplification step in the detection kit. This is although paid by a slightly increased diffuse background staining. In general, both automated platforms are widely used in routine pathology and reveal sufficient and robust staining for many different antibodies.

Bioinformatics research is ongoing to generate software tools for automated analysis of TMA localization data and XLM-based standardized data capture and transfer [31]. As presented on Figure 2, our hybrid C/TMAs are likely to be amenable to automated localization software. Further, markers such as Ber-EP4 or TTF-1 seem to be suitable for automated quantitative intensity measurements such as AQUA [32-34] or automated image texture analysis [35], due to homogeneous surface staining and absence of co-expressing background inflammatory cells. However, such techniques would be difficult to perform in case of ERCC1 (c.f. Figure 3). Also, parallel protein analysis by immunoblot or mRNA techniques would not alleviate the problem.

In the original paper of ERCC1 IHC on human FFPE tissue of NSCLC patients, the Mab 8F1 was used [4]. The specificity of this antibody was although recently challenged [15,16], since 8F1 stained a second spurious band on immunoblots from human fibroblasts but not HeLa cervical carcinoma cells and could not discriminate between ERCC1-positive and negative fibroblasts on immunofluorescence. However, 8F1 confidently detected His-tagged purified ERCC1. In reply, the authors of the first NSCLC study demonstrated that in the HeLa and the A549 lung adenocarcinoma cell lines, one major band of 36 kD was observed on immunoblot using 8F1 and this band disappeared after siRNA-mediated depletion [11]. In this study, both 8F1 and D-10 homogeneously and robustly stained the whole nuclear surface. No intranuclear compartmentalization was observed apart from omission of nucleoli or nuclear invaginations. However, D-10 showed unspecific background staining at the plasma membrane and in intracellular mucin vacuoles and was generally weaker on same protocols.

## Conclusions

In summary, cell block cytology microarrays (CMA) are suitable for investigations of relevant clinical biomarkers and can be mixed with TMA's to yield C/TMA hybrids. On the two automated IHC/ICC platforms Ventana Benchmark^® ^and Leica Bond^®^, the anti-ERCC1 antibody 8F1 performed superior compared to D-10 in terms of staining quality and restriction to the nuclear compartment.

## Competing interests

The authors declare that they have no competing interests.

## Authors' contributions

AS carried out the immunochemical scoring, performed statistical analysis and drafted the manuscript together with HM. SKH and BB diagnosed patients and assembled the cohort. SB carried out the immunochemistry, MS manufactured the C/TMAs. All authors read and approved the final manuscript.

## References

[B1] de Laat WL Jaspers NG Hoeijmakers JH Molecular mechanism of nucleotide excision repair Genes Dev 1999 13 768 785 10.1101/gad.13.7.768 10197977

[B2] Friedberg EC How nucleotide excision repair protects against cancer Nat Rev Cancer 2001 1 22 33 10.1038/35094000 11900249

[B3] Martin LP Hamilton TC Schilder RJ Platinum resistance: the role of DNA repair pathways Clin Cancer Res 2008 14 1291 1295 10.1158/1078-0432.CCR-07-2238 18316546

[B4] Olaussen KA Dunant A Fouret P Brambilla E Andre F Haddad V DNA repair by ERCC1 in non-small-cell lung cancer and cisplatin-based adjuvant chemotherapy N Engl J Med 2006 355 983 91 10.1056/NEJMoa060570 16957145

[B5] Soria JC ERCC1-tailored chemotherapy in lung cancer: the first prospective randomized trial J Clin Oncol 2007 25 2648 2649 10.1200/JCO.2007.11.3167 17602070

[B6] Schiller JH Harrington D Belani CP Langer C Sandler A Krook J Comparison of four chemotherapy regimens for advanced non-small-cell lung cancer N Engl J Med 2002 346 92 98 10.1056/NEJMoa011954 11784875

[B7] Fossella F Pereira JR von Pawel J Pluzanska A Gorbounova V Kaukel E Randomized, multinational, phase III study of docetaxel plus platinum combinations versus vinorelbine plus cisplatin for advanced non-small-cell lung cancer: the TAX 326 study group J Clin Oncol 2003 21 3016 3024 10.1200/JCO.2003.12.046 12837811

[B8] Vilmar A Sørensen JB Excision repair cross-complementation group 1 (ERCC1) in platinum-based treatment of non-small cell lung cancer with special emphasis on carboplatin: A review of current literature Lung Cancer 2009 64 131 139 10.1016/j.lungcan.2008.08.006 18804893

[B9] Gossage L Madhusudan S Current status of excision repair cross complementing-group 1 (ERCC1) in cancer Cancer Treat Rev 2007 33 565 577 10.1016/j.ctrv.2007.07.001 17707593

[B10] Booton R Ward T Ashcroft L Morris J Heighway J Thatcher N ERCC1 mRNA expression is not associated with response and survival after platinum-based chemotherapy regimens in advanced non-small cell lung cancer J Thorac Oncol 2007 2 902 906 10.1097/JTO.0b013e318155a637 17909351

[B11] Olaussen KA Fouret P Kroemer G ERCC1-specific immunostaining in non-small-cell lung cancer N Engl J Med 2007 357 1559 1561 10.1056/NEJMc072007 17928611

[B12] Zheng Z Chen T Li X Haura E Sharma A Bepler G DNA synthesis and repair genes RRM1 and ERCC1 in lung cancer N Engl J Med 2007 356 800 808 10.1056/NEJMoa065411 17314339

[B13] Lee HW Han JH Kim JH Lee MH Jeong SH Kang SY Expression of excision repair cross-complementation group 1 protein predicts poor outcome in patients with small cell lung cancer Lung Cancer 2007 59 95 104 17889401 10.1016/j.lungcan.2007.07.023

[B14] Kwon HC Roh MS Oh SY Kim SH Kim MC Kim JS Prognostic value of expression of ERCC1, thymidylate synthase, and glutathione S-transferase P1 for 5-fluorouracil/oxaliplatin chemotherapy in advanced gastric cancer Ann Oncol 2007 18 504 509 17322540 10.1093/annonc/mdl430

[B15] Niedernhofer LJ Bhagwat N Wood RD ERCC1 and non-small-cell lung cancer N Engl J Med 2007 356 2538 2540 17568038 10.1056/NEJMc070742

[B16] Bhagwat NR Roginskaya VY Acquafondata MB Dhir R Wood RD Niedernhofer LJ Immunodetection of DNA repair endonuclease ERCC1-XPF in human tissue Cancer Res 2009 69 6831 6838 10.1158/0008-5472.CAN-09-1237 19723666 PMC2739111

[B17] Wiatrowska BA Krol J Zakowski MF Large-cell neuroendocrine carcinoma of the lung: proposed criteria for cytologic diagnosis Diagn Cytopathol 2001 24 58 64 10.1002/1097-0339(200101)24:1<58::AID-DC1010>3.0.CO;2-O 11135471

[B18] Arbogast S Behnke S Opitz I Stahel R Seifert B Weder W Automated ERCC1 immunohistochemistry in non-small cell lung cancer: Comparison of anti-ERCC1 antibodies 8F1, D-10 and FL-297 Appl Immunohistochem Mol Morphol 2010 Oct. 27, E-pub 10.1097/PAI.0b013e3181f1feeb 21030862

[B19] Holm B Mellemgaard A Skov T Skov BG Different impact of excision repair cross-complementation group 1 on survival in male and female patients with inoperable non-small-cell lung cancer treated with carboplatin and gemcitabine J Clin Oncol 2009 27 4254 4259 10.1200/JCO.2008.18.8631 19667277

[B20] Bubendorf L Tissue microarrays meet cytopathology Acta Cytol 2006 50 121 122 10.1159/000325918 16610676

[B21] Pu RT Giordano TJ Michael CW Utility of cytology microarray constructed from effusion cell blocks for immunomarker validation Cancer 2008 114 300 306 10.1002/cncr.23797 18798226

[B22] Wirth GJ Schandelmaier K Smith V Burger AM Fiebig HH Microarrays of 41 human tumor cell lines for the characterization of new molecular targets: expression patterns of cathepsin B and the transferrin receptor Oncology 2006 71 86 94 10.1159/000100476 17347587

[B23] Hsu FD Nielsen TO Alkushi A Dupuis B Huntsman D Liu CL Tissue microarrays are an effective quality assurance tool for diagnostic immunohistochemistry Mod Pathol 2002 15 1374 1380 10.1097/01.MP.0000039571.02827.CE 12481020

[B24] Ueda J Iwata T Ono M Takahashi M Comparison of three cytologic preparation methods and immunocytochemistries to distinguish adenocarcinoma cells from reactive mesothelial cells in serous effusion Diagn Cytopathol 2006 34 6 10 10.1002/dc.20391 16355377

[B25] Gong Y Sun X Michael CW Attal S Williamson BA Bedrossian CW Immunocytochemistry of serous effusion specimens: a comparison of ThinPrep vs cell block Diagn Cytopathol 2003 28 1 5 10.1002/dc.10219 12508174

[B26] Fetsch PA Simsir A Brosky K Abati A Comparison of three commonly used cytologic preparations in effusion immunocytochemistry Diagn Cytopathol 2002 26 61 66 10.1002/dc.10039 11782091

[B27] Pereira TC Saad RS Liu Y Silverman JF The diagnosis of malignancy in effusion cytology: a pattern recognition approach Adv Anat Pathol 2006 13 174 184 10.1097/00125480-200607000-00004 16858151

[B28] Nathan NA Narayan E Smith MM Horn MJ Cell block cytology. Improved preparation and its efficacy in diagnostic cytology Am J Clin Pathol 2000 114 599 606 10.1309/G035-P2MM-D1TM-T5QE 11026107

[B29] Wen CH Su YC Wang SL Yang SF Chai CY Application of the microarray technique to cell blocks Acta Cytol 2007 51 42 46 10.1159/000325681 17328494

[B30] Anagnostou VK Lowery FJ Syrigos KN Cagle PT Rimm DL Quantitative evaluation of protein expression as a function of tissue microarray core diameter: is a large (1.5 mm) core better than a small (0.6 mm) core? Arch Pathol Lab Med 2010 134 613 619 20367312 10.5858/134.4.613

[B31] Dhir R Tissue microarrays: an overview Methods Mol Biol 2008 441 91 103 10.1007/978-1-60327-047-2_6 18370313

[B32] Gustavson MD Bourke-Martin B Reilly D Cregger M Williams C Mayotte J Standardization of HER2 immunohistochemistry in breast cancer by automated quantitative analysis Arch Pathol Lab Med 2009 133 1413 1419 19722747 10.5858/133.9.1413

[B33] Moeder CB Giltnane JM Moulis SP Rimm DL Quantitative, fluorescence-based in-situ assessment of protein expression Methods Mol Biol 2009 520 163 175 10.1007/978-1-60327-811-9_12 19381954

[B34] Rojo MG Bueno G Slodkowska J Review of imaging solutions for integrated quantitative immunohistochemistry in the Pathology daily practice Folia Histochem Cytobiol 2009 47 349 354 10.2478/v10042-008-0114-4 20164017

[B35] Karacali B Tozeren A Automated detection of regions of interest for tissue microarray experiments: an image texture analysis BMC Med Imaging 2007 7 2 10.1186/1471-2342-7-2 17349041 PMC1838905

